# Sharing Responsibility: Responsibility for Health Is Not a Zero-Sum Game

**DOI:** 10.1093/phe/phz012

**Published:** 2019-07-24

**Authors:** Marcel Verweij, Angus Dawson

**Affiliations:** 1 Communication, Philosophy and Technology, Department of Social Sciences, Wageningen University; 2 Sydney Health Ethics, Sydney School of Public Health, University of Sydney

## A Societal Agreement on Prevention

In late 2018, a National Prevention Agreement was signed by the government of the Netherlands, in collaboration with local governments and 70 other societal partners, representing public and private, for profit and non-profit organizations (VWS, 2018). The National Prevention Agreement focuses on three common themes related to non-communicable diseases: the prevention of overweight and obesity, of smoking and of excessive alcohol intake. The ambitions are high: the partners to the agreement commit themselves to the objectives that in 2040 no adolescents will smoke anymore; that the prevalence of overweight of persons above 20 years will have declined from 50 per cent to 38 per cent; and that not more than 5 per cent of adults drink too much alcohol. The measures include amongst others smoke-free schoolyards, a ban on in-school sale of sugared drinks and healthier food offers in company restaurants, school canteens and the creation of more attractive parks and public spaces. The opportunities for the marketing of unhealthy products at children will be constrained. Retailers will not promote alcoholic drinks with special offers that offer more than 25 per cent price reductions. The agreement foresees tax increases on tobacco products: in a few years cigarettes will cost €10 per pack.

Prominent examples of possible policies that were not proposed are sugar or junk food taxes, and a drastic restriction of places where tobacco products could be sold. NGOs and public health professionals also complain that the agreement largely consists of voluntary steps that will not be enforced; a limitation that is arguably due to the fact that the government was keen to keep all parties—notably private companies such as the large retailers—on board. The RIVM (National Institute of Public Health and Environment) does not expect that the ambitions will be realized given the measures that are agreed upon (RIVM, 2018). However, the RIVM will have the task to monitor the program and the progress that will be made, and the idea is that more strict measures will be taken if necessary. The prevention agreement is seen as expressing a collective responsibility for health that is shared by government, societal partners and citizens—although the latter were only represented via the democratically elected government and ultimately by parliament.

## Is Responsibility for Health a ‘Zero-Sum Game’?

Notwithstanding the critique that one can have of the contents of the agreement, the current government has clearly chosen a different road than the previous Minister of Health, who was highly reluctant to expand the role of government in preventing what might be called ‘lifestyle’ diseases. In her 2011 policy brief (VWS, 2011), she claimed that healthy lifestyles had been considered for too long a responsibility of professionals and government, with a focus on what individuals should (not) do, leaving too little responsibility for individual persons themselves. Responsibility for healthy behaviour should be given back to where it belonged, that is, to each individual for herself. This idea fits, of course, clearly in a liberal discourse that rejects paternalism. For liberals and many others, the vice of paternalism is not just that it involves constraints on liberty; state paternalism is especially objectionable as far as it involves taking responsibility for someone’s wellbeing rather than allowing that person be responsible for her own life. That would be like treating that person as a child that cannot care for herself, and seeing the state as a parent (or worse: nanny) that takes the responsibility that a child-like citizen lacks. On the other hand, respecting a competent individual that is capable of autonomous choice would involve emphasizing her own individual responsibility for health and thus reducing the sphere of responsibility of the state. (Of course, there is far more to say about paternalism in public health (Nys, 2008; Wilson, 2011)).

This way of reasoning is not uncommon in antipaternalist thought. It apparently presupposes a view of responsibility for health as being a ‘zero-sum game’ (Grill and Nihlén Fahlquist, 2012; Verweij, 2014): if one party assumes more responsibility (notably government or other societal organizations) this would come at the cost of others that also have (or should have) responsibility—as if responsibility is like a pie that is to be divided between people that each will have a smaller or larger share. Does this assumption hold? Discussing this issue can offer some clarity about what it means for various parties to share responsibility, and in that way it also may help to clarify the normative implications of seeing health as a collective, shared responsibility either in the way that the current Dutch prevention agreement suggests or in other ways that are implied or claimed in public health theory and practice.

The problem with discussions about ‘responsibility’ is that the term can refer to various concepts that may partly overlap or relate to one another but are clearly distinct. Hence, one attempt to clarify discussions about responsibility for health is to tease out these different meanings and argue which of these concepts should be central to specific policies, communications or normative arguments. This is what some of the papers in this issue of *Public Health Ethics* aim to do—notably the paper by Brown and colleagues. Offering an encompassing systematic analysis of responsibility concepts goes beyond the scope of this editorial, but one can at least identify differences between responsibility as being backward-looking or forward-looking (cf. Nihlén Fahlquist, 2006); as accountability or attributability (cf. Scanlon, 1998; Watson, 2004); as moral or causal responsibility; or as a matter of prudence (Brown *et al.*, 2019). Moreover, the term can refer to a task, to an obligation or to a virtue (Nihlén Fahlquist, forthcoming).

Arguably, for some of these concepts, the presumption of responsibility as a ‘zero-sum game’ might hold. It would sometimes make sense if we are talking about subtasks that are assigned to different parties to achieve a specific goal. If some do more, they might do others’ jobs, and thus there would be less for those others to do. The presumption of responsibility as a ‘zero-sum game’ might also hold in cases of causal responsibility: the larger the causal role of one determinant might imply that other factors are less important for a certain event to occur. Analogously, if moral responsibility for a certain event (for example, me getting drunk) can be attributed to different agents (myself, the bartender, my friends who encouraged me to take another drink), who, looking back, all contributed to the event, then if some played only a very small part, others will be more responsible, and vice versa.

In our view, the issue of paternalism and individual versus government responsibility for health is primarily a matter of *forward-looking* responsibility that focuses on the normative reasons government and citizens have to promote and protect health. In this context, there is no ground for assuming that a stronger role for government would imply a smaller responsibility for individuals, or vice versa. This is because ethical reasons and objectives of the state to promote healthy behaviour are different from individual persons’ reasons to take care of their own health. For example, taking care of my own nutrition and health is advisable for prudential and moral reasons: it serves my own good and it protects my ability to care for my family and fulfil other duties. These prudential and moral reasons for making healthy choices can be very strong, but that does not downplay the moral reasons that the state has to promote healthy nutrition as well. From a public health perspective, it would actually be unfair to leave all responsibility for healthy nutrition to individual citizens themselves. It might be fair if everyone had an equal chance to a healthy way of living. But in fact huge inequalities exist within populations and therefore the state has reasons of justice to take on responsibility for healthy nutrition. Other normative grounds for health policies include the prevention of harm to others or the protection of public goods within a solidaristic health system (Davies and Savulescu, 2019). Promoting healthy nutrition via information, education or nudges will clearly not push aside or diminish the reasons that individuals have to care about their own nutrition. But even if the sale of certain products would be banned (e.g. supersized soda-drinks, as in the controversial New York proposal by Michael Bloomberg) this is not making the responsibility of individuals smaller—they still have their own reasons for being concerned about their health, and they still have ample opportunities to take care of, or neglect their own health. In the context of the forward-looking responsibilities of individual citizens and the government—but also for societal organizations and private companies—responsibility for health is not a ‘zero-sum game’. Different parties have different moral and prudential grounds as well as variable opportunities for caring about healthy behaviour. These reasons do not necessarily compete, or overtake one another.

## Sharing Responsibility for Prevention

Acknowledging that responsibility for health is not a ‘zero-sum game’—not even if the focus is on healthy or unhealthy behaviour—can help take out the sting of some criticism of governmental public health activities. Public health authorities that aim at creating better social conditions for health, or at a reduction of health inequalities, or at less harmful environments, are not robbing individual citizens of their responsibility. They are taking their own public responsibility and do not usurp a citizen’s individual responsibility for health.

Acknowledging that there are different moral grounds for responsibility for (promoting and protecting) health, and that different actors can have different moral reasons for contributing to health, may also shed some light on the normativity of the prevention agreement in the Netherlands or on similar public–private partnerships for health.

One interpretation of the agreement would be that it forms the basis for each party’s responsibility, and that government, and public and private partners have agreed who should do what (hence a division of tasks) to meet the overall ambitions of the agreement. On this interpretation their shared responsibility looks like a ‘zero-sum game’ indeed; at least if the number and scope of tasks is limited and as far as doing more than agreed implies taking over the tasks of others. This interpretation however does not make much sense. First because there is arguably much more that every party can do to promote health without taking away possibilities for others. More importantly, all parties have their own moral grounds for promoting health and refraining from contributing to ill-health. These do not collide or compete, and, moreover, they serve as the basis for each actor’s responsibility—a basis that is independent of the strength, validity or desirability of the agreement as such. Much has been written, particularly in this journal, on the various ethical grounds for the state to promote health. But these justifications for public health activities do not negate moral principles underlying responsibilities of, for example, private companies to promote and protect health as well: obligations of industry and retailers to refrain from harming people by selling products that undermine health; to mitigate structural inequities (Tempels *et al.*, 2017); to protect and strengthen autonomous choice (nudges for health; refraining from all-too-persuasive strategies to sell energy-dense foods or drinks; abandoning child-marketing); or to ensure the safety and nutritious quality of food; etc.

If we acknowledge that governments as well as public and private organizations all have compelling ethical reasons to promote and protect health, this implies that it is not so much a ‘national agreement’ or public–private partnership that defines what the scope of their responsibility is. A societal agreement like the one signed in the Netherlands should be seen as *expressing*, not as underpinning or defining their responsibility. A relevant implication is that responsibility may well go beyond what is agreed. Another implication is that these ‘shared responsibilities’ are not mutually conditional. If some party at some point steps out of the agreement this will not affect their responsibility or that of the remaining parties. Their responsibility for health is warranted independent of the agreement they have made.

## Responsibility in *Public Health Ethics*

In this issue of *Public Health Ethics* we have collected a variety of papers, dealing with different senses, dimensions and implications of responsibility for health. Neil Levy argues that discussions about individual responsibility for health should take into account that capacities and circumstances necessary for responsible choice are distributed unequally, and that discussions should focus more on holding those responsible who determine the ways in which capacities and circumstances are distributed (Levy, 2019). Rebecca Brown and colleagues also criticize the emphasis on individual moral responsibility for health in health policies. Instead, they propose to shift the attention to a prudential (hence non-moral) understanding of responsibility (Brown *et al.*, 2019). Kathryn Mackay reflects on their proposal and indicates a potential weakness in their view (Mackay, 2019). The connection between responsibility and solidarity is discussed by Davies and Savulescu: they argue that health care systems that are grounded in solidarity, under certain conditions, have the right to penalize some users who are responsible for their poor health (Davies and Savulescu, 2019). Davies and Savulescu’s normative analysis offers an interesting counterpoint to Gloria Traina’s empirical study of Norwegian citizens’ views of personal responsibility for health. The results of their survey suggest that a significant support for social responsibility does not exclude a strong support for personal health responsibility (Traina *et al.*, 2019).

We look forward to receiving more work on this complex topic. A systematic analysis of the different concepts of responsibility for health, overseeing their implications for specific discussions in public health ethics would be especially welcome.
